# Loss of DDHD2, whose mutation causes spastic paraplegia, promotes reactive oxygen species generation and apoptosis

**DOI:** 10.1038/s41419-018-0815-3

**Published:** 2018-07-23

**Authors:** Tomohiro Maruyama, Takashi Baba, Yuki Maemoto, Chikako Hara-Miyauchi, Minami Hasegawa-Ogawa, Hirotaka James Okano, Yuki Enda, Kei Matsumoto, Nagisa Arimitsu, Kazuki Nakao, Hiroshi Hamamoto, Kazuhisa Sekimizu, Takayo Ohto-Nakanishi, Hiroki Nakanishi, Takeshi Tokuyama, Shigeru Yanagi, Mitsuo Tagaya, Katsuko Tani

**Affiliations:** 1 School of Life Sciences, Tokyo University of Pharmacy and Life Sciences, Hachioji Tokyo, 192-0392 Japan; 2 Division of Regenerative Medicine, Jikei University School of Medicine, 3-25-8 Nishi-Shimbashi, Minato-ku Tokyo, 105-8461 Japan; 3 Laboratory of Animal Resources, Center for Disease Biology and Integrative Medicine, Graduate School of Medicine, The University of Tokyo, Tokyo, 113-0033 Japan; 4 Graduate School of Pharmaceutical Sciences, The University of Tokyo, Tokyo, 113-0033 Japan; 5 Japan Lipid Technologies LLC, Akita, Akita, 010-0825 Japan; 6 Research Center for Biosignaling, Akita University, Akita, 010-8543 Japan; 7 Present Address: Section on Molecular Signal Transduction, Program for Developmental Neuroscience, Eunice Kennedy Shriver NICHD, National Institutes of Health, Bethesda, MD 20892 USA; 8 Present Address: Department of Immunology and Medicine, St. Marianna University School of Medicine, 2-16-1 Sugao, Miyamae, Kawasaki, Kanagawa 216-8511 Japan; 9 Present Address: Teikyo University Institute of Medical Mycology, 359 Otsuka, Hachioji Tokyo, 192-0395 Japan

## Abstract

DDHD2/KIAA0725p is a mammalian intracellular phospholipase A_1_ that exhibits phospholipase and lipase activities. Mutation of the *DDHD2* gene causes hereditary spastic paraplegia (SPG54), an inherited neurological disorder characterized by lower limb spasticity and weakness. Although previous studies demonstrated lipid droplet accumulation in the brains of SPG54 patients and *DDHD2* knockout mice, the cause of SPG54 remains elusive. Here, we show that ablation of DDHD2 in mice induces age-dependent apoptosis of motor neurons in the spinal cord. In vitro, motor neurons and embryonic fibroblasts from *DDHD2* knockout mice fail to survive and are susceptible to apoptotic stimuli. Chemical and probe-based analysis revealed a substantial decrease in cardiolipin content and an increase in reactive oxygen species generation in DDHD2 knockout cells. Reactive oxygen species production in DDHD2 knockout cells was reversed by the expression of wild-type DDHD2, but not by an active-site DDHD2 mutant, DDHD2 mutants related to hereditary spastic paraplegia, or DDHD1, another member of the intracellular phospholipase A_1_ family whose mutation also causes spastic paraplegia (SPG28). Our results demonstrate the protective role of DDHD2 for mitochondrial integrity and provide a clue to the pathogenic mechanism of SPG54.

## Introduction

Hereditary spastic paraplegia (HSP) is a diverse group of neurological disorders characterized by lower limb spasticity and weakness^1,2^. These symptoms are due to length-dependent axonopathy of corticospinal motor neurons, sometimes associated with a loss of cortical neurons and anterior horn cells^3^. The severity of HSP is variable, and the age at onset ranges from early childhood to late in life. To date, nearly 80 genes or loci have been identified and numbered (spastic paraplegia (SPG) 1–79). HSP-causing genes encode proteins involved in axon pathfinding, cytoskeleton organization, membrane trafficking, endoplasmic reticulum (ER) shaping, and mitochondrial functions^1,2^.

The intracellular phospholipase A_1_ (PLA_1_) protein family is a relatively recently discovered lipid-metabolizing enzyme family, and characterized by the presence of the short lipase active-site sequence Gly-X-Ser-X-Gly (X represents any amino acid) and a C-terminal DDHD (named after the presence of conserved three Asp residues and one His residue) domain. This family in mammals consists of three members^4^: phosphatidic acid-preferring PLA_1_/DDHD1^5^, p125/Sec23IP^6^, and KIAA0725p/DDHD2^7^. DDHD1 is highly expressed in brain and testis^5,8^, and is involved in sperm formation^9^. Mutations in the *DDHD1* gene have been reported to cause HSP^10^, but no obvious SPG symptoms were observed in *DDHD1* knockout (KO) mice^9^. Sec23IP is localized in ER exit sites^11^, and the KO mice also exhibit a deficiency in spermiogenesis^12^. Mutations in the *DDHD2* gene also cause HSP^13–17^. Although DDHD1 is cytosolic^8,9^, DDHD2 is localized in both the cytosol and membranes including the Golgi apparatus^7,18^, and perhaps the ER^7^. For membrane binding, both lipase activity^19^ and a sterile alpha motif (SAM) domain flanked by the DDHD domain^20^ are important. Interestingly, treatment with CI-976, a lysophospholipid acyltransferase antagonist, causes the redistribution of cytosolic DDHD2 to tubular structures in close vicinity to mitochondria^21^.

Patients with *DDHD2* mutations are characterized by a thin corpus callosum^13–17^ and lipid accumulation in the brain, as detected on cerebral magnetic resonance spectroscopy^13,17^. The HSP phenotype and lipid accumulation were observed in *DDHD2* KO mice^22^. Mass spectrometry-based lipidomics revealed that DDHD2 regulates brain triacylglycerol (TAG) levels, and that recombinant DDHD2 displayed TAG lipase activity^22^. Other studies demonstrated that DDHD2 exhibits diacylglycerol lipase activity^23,24^. Although lipid droplet (LD) accumulation in the brain of *DDHD2* KO mice is likely due to the lack of lipase activity derived from DDHD2, the reason why age-dependent motor neuron degeneration occurs in SPG remains unknown. Here we show that DDHD2 ablation induces reactive oxygen species (ROS) production in mitochondria, thereby leading to apoptosis. Expression of wild-type (WT) DDHD2, but not the active-site mutant or mutants related to SPG, in DDHD2-deficient cells prevents ROS production and facilitates their consumption.

## Results

### Loss of motor neurons in the spinal cords of *DDHD2* KO mice

To reveal the physiological function of DDHD2, we generated *DDHD2* KO mice. Using a targeting vector that contains exons 8 and 9 flanked by two *loxP* sites (Supplementary Figure 1a), we obtained targeted cell lines, and then generated chimeric, flox, and heterozygous and homozygous KO mice, as described under Materials and methods. Southern and Western blotting (WB) demonstrated the loss of exons 8 and 9 in the *DDHD2* gene and DDHD2 protein, respectively, in the obtained mice (Supplementary Figure 1b). *DDHD2* KO mice exhibited a paw clasping response (Supplementary Figure 1c) and reduced hind limb extension behavior (Supplementary Figure 1d) in an age-dependent manner. Moreover, reduced stride lengths were observed in 6-month-old KO mice (Supplementary Figure 1e). These are typical features of SPG^1,2^.

**Fig. 1:**
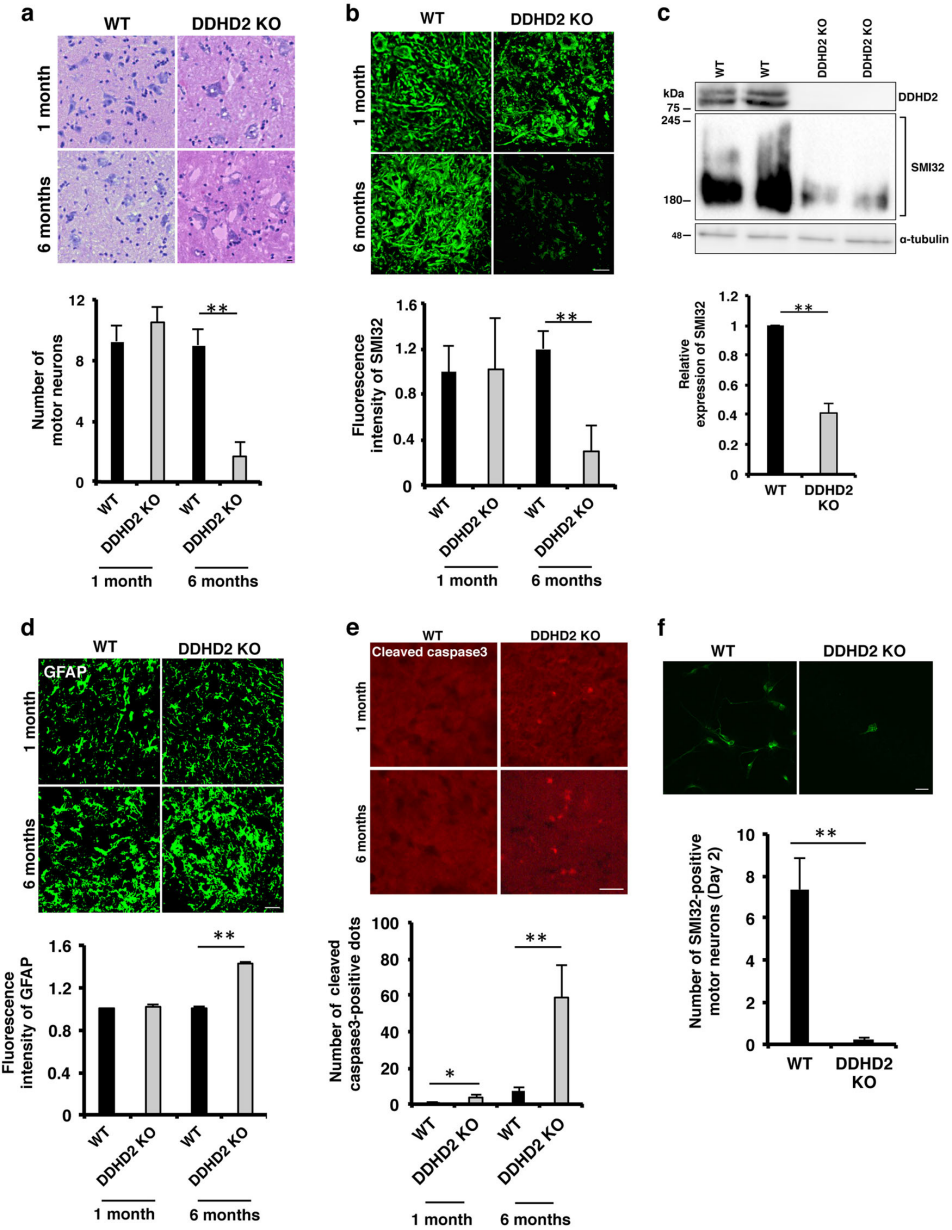
Loss of DDHD2 induces motor neuron degeneration in the spinal cord. **a** Hematoxylin and eosin staining of lumbar spinal cords of WT and *DDHD2* KO mice The motor neurons were counted. Values are means ± SEM (*N* = 3). **b**, **d**, **e** Photomicrographs of cross-sections of the lumbar spinal cords of WT and *DDHD2* KO mice labeled with an antibody against SMI32 (**b**), GFAP (**d**), or cleaved caspase3 (**e**). The total fluorescence intensity or the total number of cleaved caspase3-positive cells in each spinal cord section was determined. Six sections were analyzed in each mouse, and three mice were used for each experiment. Data represent means ± SEM (*N* = 3). **c** Spinal cord lysates (20 µg) of WT and *DDHD2* KO mice at 6 months of age were analyzed by WB with antibodies against DDHD2, SMI32, and α-tubulin. The intensity of immunoreactive signals was quantified, and the SMI32 level in KO mice relative to that in WT mice is expressed as means ± SEM (*N* = 3). **f** Primary motor neurons from the spinal cords of WT and *DDHD2* KO mice were cultured for 2 days, and then immunostained with an anti-SMI32 antibody. The number of surviving SMI32-positive motor neurons in an arbitrarily chosen microscopic field with a size of 212 × 212 μm^2^ was determined. Six fields were analyzed in each experiment. Data represent means ± SEM (*N* = 3). The scale bars for whole cell panels are 10 μm. *P < 0.05, **P < 0.01, Student’s *t*-test

Hematoxylin and eosin staining of the lumbar spinal cords of *DDHD2* KO mice revealed the loss of motor neurons at 6 months, but not 1 month, of age (Fig. 1a). However, vacuoles were seen even in 1-month-old mice (Fig. 1a). Sudan III staining showed the accumulation of neutral lipids in the spinal cords of juvenile *DDHD2* KO mice, but not of WT mice (data not shown), suggesting that LDs start to accumulate in juvenile *DDHD2* KO mice. Immunostaining and WB demonstrated a marked reduction in cells positive for SMI32, a motor neuron marker^25^, in the lumbar spinal cords of 6-month-old *DDHD2* KO mice (Fig. 1b, c). It should be noted that there was no substantial difference in the number of SMI32-positive cells between WT and *DDHD2* KO mice at the age of 1 month. Concomitantly with the loss of motor neurons, the number of astrocytes positive for GFAP^26^ increased (alternatively, astrocytes were activated and their size increased) (Fig. 1d). Consistent with the reduction of motor neurons, many apoptotic cells visualized as the formation of cleaved caspase3 were observed in the spinal cords of *DDHD2* KO mice at the age of 6 months, but not 1 month (Fig. 1e).

We investigated the viability of motor neurons in *DDHD2* KO mice embryos. Neurons were dissected from the spinal cords and brains of embryos and then cultured. At day 2, most spinal cord motor neurons from *DDHD2* KO mice had died, although those from WT mice survived well (Fig. 1f). On the other hand, neurons in the motor cortex of *DDHD2* KO mice survived, similar to those from the WT mice (Supplementary Figure 1f). These results suggest that the DDHD2 deficiency causes degeneration of motor neurons in the spinal cord, not the motor cortex, in an age-dependent manner.

### Loss of DDHD2 increased sensitivity to apoptosis in vivo and in vitro

**Fig. 2:**
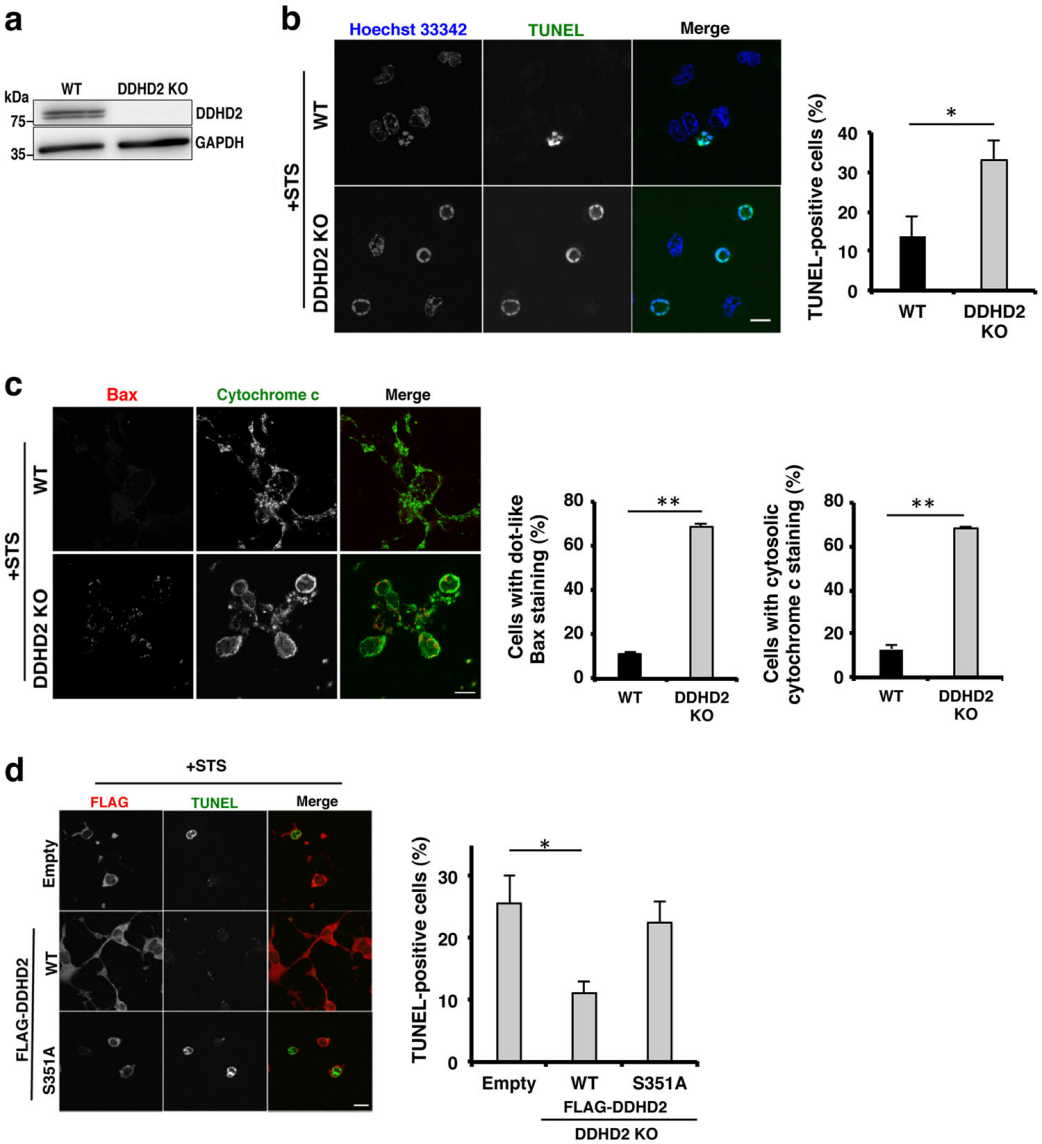
Loss of DDHD2 increases apoptosis sensitivity of MEFs. **a** Lysates (10 µg) of WT and *DDHD2* KO MEFs were analyzed by WB with antibodies against DDHD2 and GAPDH. **b**, **c** WT and *DDHD2 KO* MEFs were treated with 1 µM STS for 8 h, followed by staining with TUNEL and Hoechst 33342 (**b**), or antibodies against Bax and cytochrome c (**c**). The number of TUNEL-positive cells or cells exhibiting dot-like Bax staining or cytosolic cytochrome c staining was determined. **d** *DDHD2* KO MEFs were transfected with a plasmid encoding FLAG (Empty), FLAG-DDHD2 WT, or FLAG-DDHD2-S351A. At 24 h after transfection, STS was added to a final concentration of 1 µM, and the cells were incubated for 8 h, and then subjected to staining with TUNEL and an anti-FLAG antibody. The number of TUNEL-positive cells was determined. No significant difference in the percentage of TUNEL-positive cells was observed between cells transfected with FLAG and FLAG-DDHD2-S351A. Data represent means ± SEM for nine (**b**) and three (**c**, **d**) independent experiments, respectively. The scale bars for whole cell panels are 10 μm. *P < 0.05, **P < 0.01, Student’s *t*-test

Given that motor neurons in the spinal cords of *DDHD2* KO mice not only underwent apoptosis in an age-dependent manner, but also could not survive in vitro even at the embryonic stage, we reasoned that motor neurons of *DDHD2* KO mice might suffer from some stresses due to DDHD2 ablation. Moreover, such stresses might occur not only for motor neurons but also for other cells. Indeed, mouse embryo fibroblasts (MEFs) from *DDHD2* KO mice could not proliferate well (data not shown). We therefore generated immortalized MEFs by introducing large T antigen using a retrovirus expression system. Unless otherwise described, we used immortalized MEFs for further studies. We first confirmed that DDHD2 protein was expressed in WT MEFs, but not *DDHD2* KO MEFs (Fig. 2a). Next, we examined the sensitivity of MEFs to staurosporine (STS), a well-known apoptosis inducer that preferentially activates Bax^27^. On treatment with STS, the percentage of TUNEL-positive cells increased more than two-fold in comparison to WT cells (Fig. 2b). Consistent with the results of the TUNEL analysis, the percentages of cells with dot-like staining for the proapoptotic protein Bax and cytosolic staining for cytochrome c were much higher in *DDHD2* KO MEFs than WT cells (Fig. 2c). Importantly, the enhancement of STS-induced apoptosis in *DDHD2* KO MEFs was reversed by the expression of FLAG-DDHD2 WT, but not the S351A mutant, in which active-site residue Ser351 was replaced by Ala (Fig. 2d), suggesting that the enzymatic activity of DDHD2 is important for cell survival. To determine whether the enhanced sensitivity to apoptosis is not limited to MEFs, we used U2OS cells. We knocked down DDHD2 in U2OS cells with two short interfering RNAs (siRNAs), and then examined the sensitivity of DDHD2-depleted cells to STS and H_2_O_2_. Upon knockdown of DDHD2, the percentages of TUNEL-positive cells and cleaved caspase3-positive cells in response to STS and H_2_O_2_ treatment, respectively, substantially increased (Supplementary Figures 2a and b). These results suggest that loss of DDHD2 causes stresses not only for motor neurons but also for cells in general.

### Accumulation of ROS in *DDHD2* KO and silenced cells

**Fig. 3:**
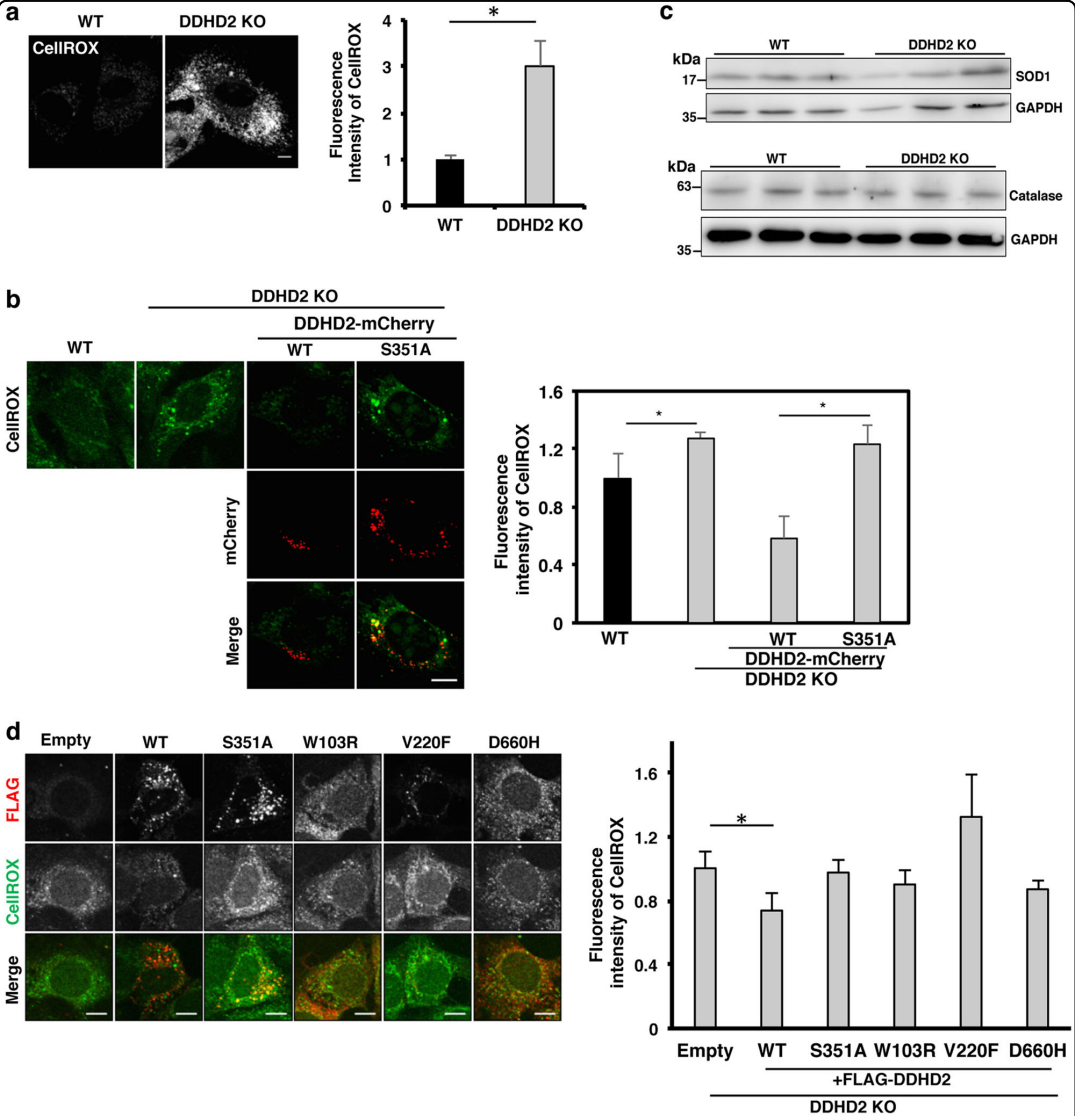
ROS accumulation in *DDHD2* KO MEFs. **a** Non-immortalized WT and *DDHD2* KO MEFs were incubated with 2.5 µM CellROX Green for 30 min, and then analyzed by IF microscopy. The graph shows the ratio of the CellROX Green intensity of *DDHD2* KO MEFs to that of WT MEFs. **b** WT MEFs, *DDHD2* KO MEFs, and *DDHD2* KO MEFs with stable expression of DDHD2-WT-mCherry or DDHD2-S351A-mCherry were incubated with 2.5 µM CellROX Green for 30 min, and then analyzed. The graph shows the ratio of the CellROX Green intensity of *DDHD2* KO MEFs or those with stable expression of DDHD2 constructs to that of WT cells. **c** Lysates (20 µg) of WT and *DDHD2* KO MEFs were analyzed by WB with antibodies against SOD1, catalase, and GAPDH. Three different preparations were analyzed. **d** *DDHD2* KO MEFs were transfected with a plasmid encoding FLAG (Empty), FLAG-DDHD2 WT, FLAG-DDHD2-S351A, FLAG-DDHD2-W103R, FLAG-DDHD2-V220F, or FLAG-DDHD2-D660H. At 24 h after transfection, the cells were incubated with 2.5 µM CellROX Green for 30 min and analyzed. The fluorescence intensity of CellROX was quantified and is expressed as the ratio relative to that in WT MEFs. No significant difference in the intensity of CellROX staining was observed between cells transfected with FLAG and SPG-associated mutant constructs. The scale bars for whole cell panels are 10 μm. Data represent means ± SEM for four (**a**) and three (**b** and **d**) independent experiments, respectively. *P < 0.05, Student’s *t*-test

Increased levels of ROS are known to cause damage to lipids, proteins, and DNA, often leading to cell death. Motor neurons are quite vulnerable to oxidative stress, as evidenced by the fact that mutations in superoxide dismutase 1, an enzyme that consumes superoxide radicals, induces amyotrophic lateral sclerosis^28^. Therefore, we next examined whether loss of DDHD2 increased the level of intracellular ROS. We used CellROX, a fluorogenic probe that can detect ROS in both live and fixed cells. We first examined MEFs without immortalization treatment. The intensity of CellROX fluorescence was three-fold higher in *DDHD2 KO* MEFs than WT MEFs (Fig. 3a). In the case of immortalized MEFs, the intensity of CellROX fluorescence in *DDHD2* KO MEFs was ∼25% higher than that in WT MEFs (Fig. 3b). However, the expression levels of enzymes responsible for the clearance of ROS, such as superoxide dismutase 1 and catalase were not significantly affected (Fig. 3c). ROS production was also detected in siRNA-silenced U2OS cells (Supplementary Figure 3a). It should be noted that the stable expression of DDHD2-WT-mCherry in *DDHD2* KO MEFs caused a 62% reduction in the intensity of CellROX staining, whereas no reduction was observed when the S351A mutant was expressed (Fig. 3b). Expression of the FLAG-DDHD2 mutants related to SPG also failed to decrease the intensity of CellROX staining (Fig. 3d). Moreover, stable expression of DDHD1, another member of the intracellular PLA_1_ family, could not reverse the enhanced CellROX staining (Supplementary Figure 4), suggesting that the two family members play different roles.

### Mitochondrial ROS production in *DDHD2* KO cells impairs mitochondrial function

**Fig. 4:**
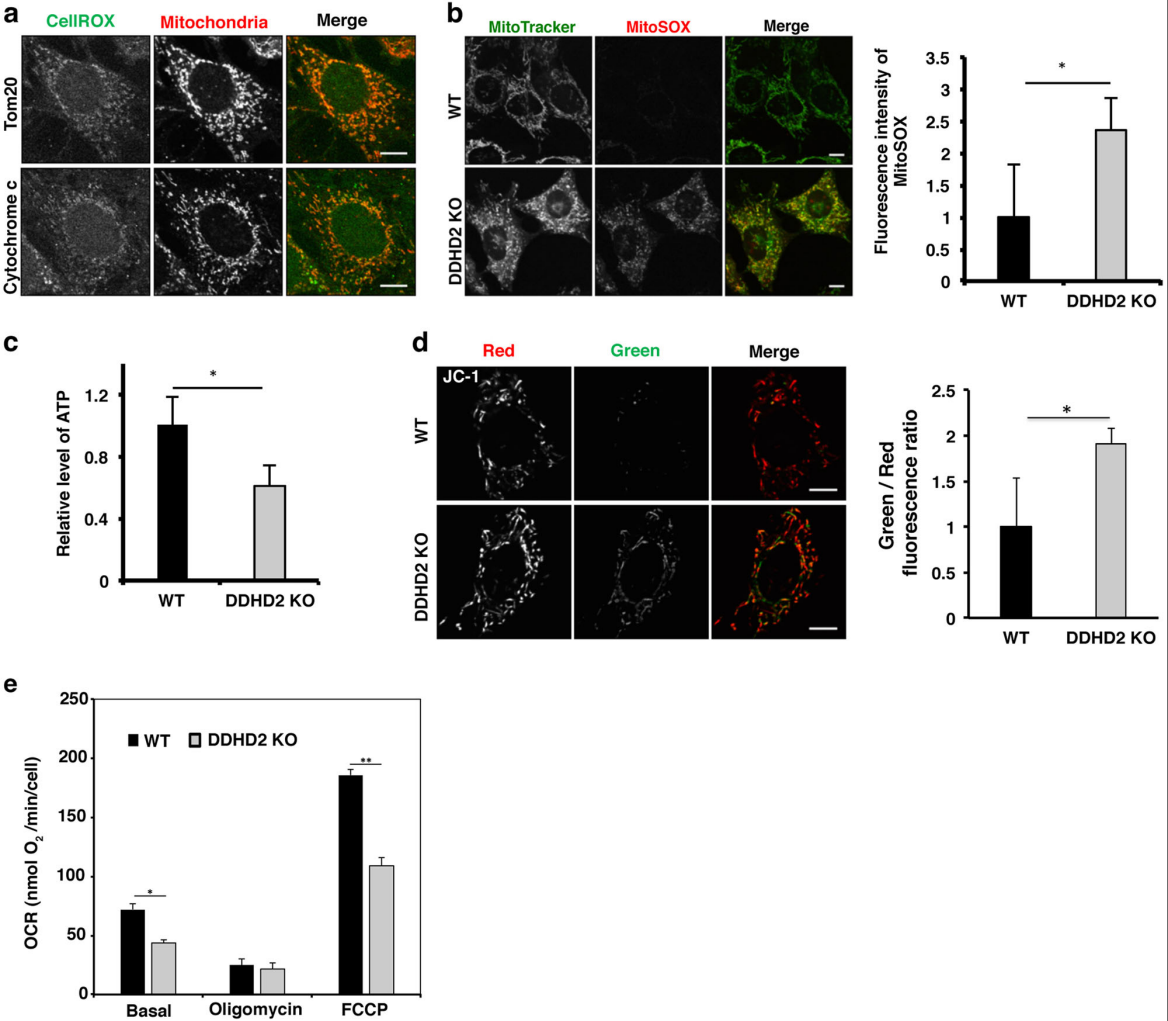
Loss of DDHD2 induces mitochondrial dysfunction in MEFs. **a** *DDHD2* KO MEFs were incubated with 2.5 µM CellROX Green for 30 min, and then stained with an antibody against Tom20 or cytochrome c and analyzed by IF microscopy. **b** WT and *DDHD2* KO MEFs were incubated with 5 µM MitoSOX and 100 nM MitoTracker Green FM for 10 min, and then analyzed by IF microscopy without fixation. The fluorescence intensity of MitoSOX was quantified and is expressed as the ratio relative to that in WT MEFs. **c** The ATP contents in WT and *DDHD2* KO MEFs were measured. The graph shows the ratio of the ATP level in *DDHD2* KO MEFs to that in WT MEFs. **d** WT and *DDHD2* KO MEFs were incubated with 2 µM JC-1 for 30 min, and then analyzed by IF microscopy without fixation. The ratio of Green/Red fluorescence intensity of JC-1 was determined and normalized as to that in WT MEFs. **e** OCRs of WT and *DDHD2* KO MEFs were measured as described under Materials and methods. The scale bars for whole cell panels are 10 μm. Data represent means ± SEM for three (**b**, **d**, **e**) and five (**c**) independent experiments, respectively. *P < 0.05, **P < 0.01, Student’s *t*-test

Because the major source of ROS in cells is mitochondria, we examined whether ROS formation in the absence of DDHD2 occurs at mitochondria. *DDHD2* KO MEFs were double stained with CellROX and mitochondrial proteins (Tom20 and cytochrome c). The CellROX-positive structures almost completely overlapped with the mitochondrial proteins (Fig. 4a). We used MitoSOX, a probe that specifically detects ROS produced in mitochondria. The MitoSOX staining also overlapped with the MitoTracker Green FM staining (Fig. 4b). In *DDHD2* KO MEFs, the ability of ATP generation decreased (Fig. 4c). In parallel with this, the mitochondrial membrane potential estimated as the ratio of green to red fluorescence of JC-1 increased (Fig. 4d), and the oxygen consumption rate (OCR) decreased (Fig. 4e). In U2OS cells, the intensity of MitoSOX staining increased upon DDHD2 depletion (Supplementary Figure 3b). Consistent with the view that ROS is responsible for apoptosis, incubation of U2OS cells depleted of DDHD2 with *N*-acetylcysteine (NAC) prevented apoptosis, as visualized as prevention of caspase3 cleavage (Supplementary Figure 3c).

**Fig. 5:**
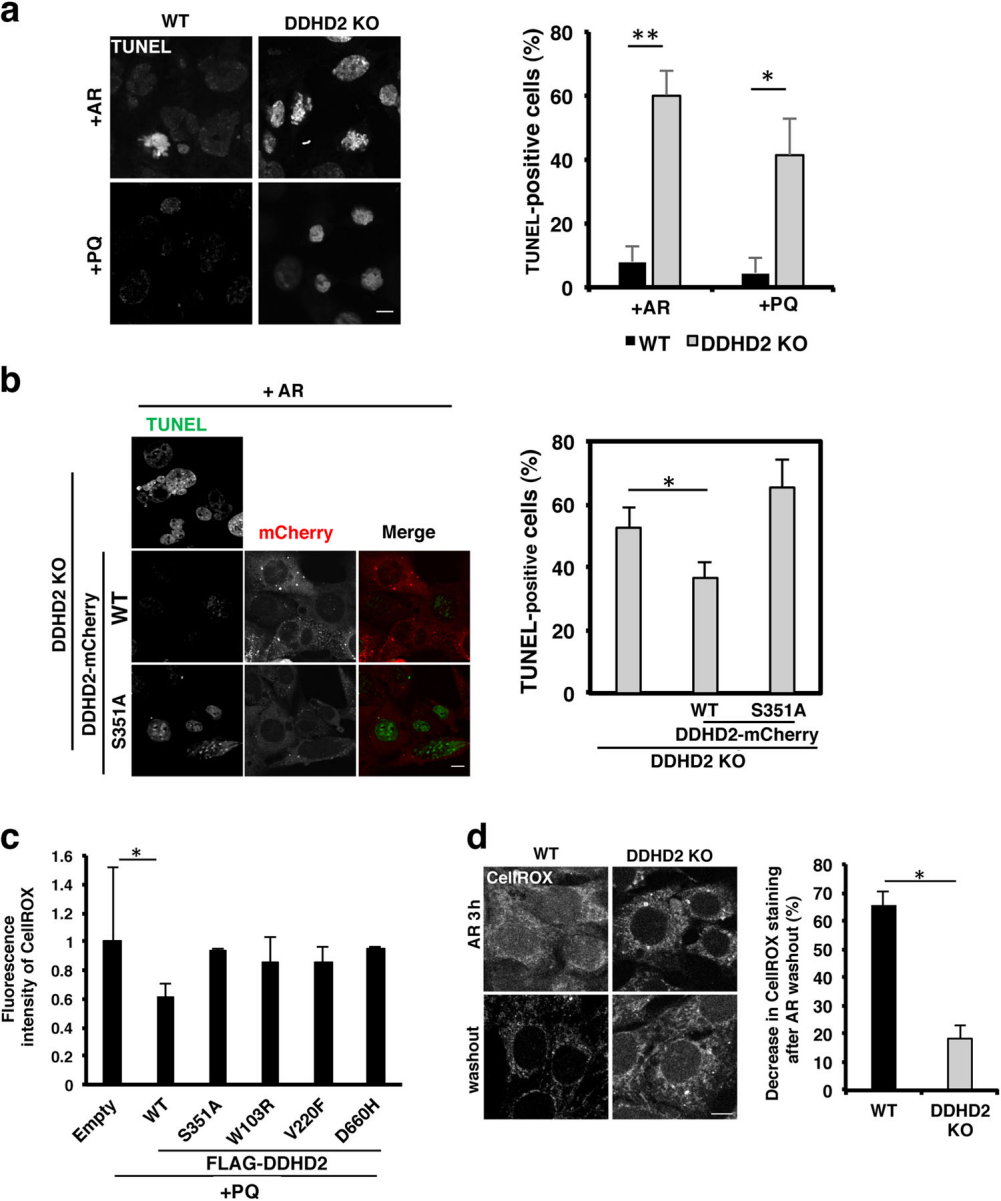
Loss of DDHD2 increases ROS-induced apoptosis sensitivity and delays ROS clearance in MEFs. **a** WT and *DDHD2* KO MEFs were treated with 2 µM antimycin A and 1 µM rotenone (AR) or 0.1 mM PQ for 16 h, followed by staining with TUNEL. The number of TUNEL-positive cells was determined. **b** *DDHD2* KO MEFs and ones with stable expression of DDHD2-WT-mCherry or DDHD2-S351A-mCherry were treated with AR for 16 h, followed by staining with TUNEL. The number of TUNEL-positive cells was determined. **c** WT MEFs were transfected with a plasmid encoding FLAG (Empty), FLAG-DDHD2 WT or one of the indicated constructs. At 24 h after transfection, the cells were treated with 0.1 mM PQ for 1 h, followed by incubation with 2.5 µM CellROX Green for 30 min and analysis. The fluorescence intensity of CellROX Green was quantified and is expressed as the ratio relative to that in MEFs with FLAG vector transfection. No significant difference in the intensity of CellROX staining was observed between cells transfected with FLAG and SPG-associated mutant constructs. **d** WT and *DDHD2* KO MEFs were incubated with 2 μM antimycin A and 1 μM rotenone (AR) for 3 h, washed, and then incubated without AR for 1 h. To detect ROS, cells were incubated with 2.5 µM CellROX Green for 30 min. The fluorescence intensity of CellROX Green relative to that observed just after 3-h AR treatment was determined. The scale bars for whole cell panels are 10 μm. Data in this figure represent means ± SEM (*N* = 3). *P < 0.05, **P < 0.01, Student’s *t*-test

We next examined whether DDHD2 can protect cells from oxidative stress. To this end, we used antimycin A-rotenone (AR) and paraquat (PQ) to produce ROS in mitochondria. First, we confirmed that *DDHD2* KO MEFs were more susceptible to AR and PQ than WT MEFs (Fig. 5a), and that this susceptibility was reversed by expression of DDHD2-WT-mCherry, but not the S351A mutant (Fig. 5b). ROS generation induced by PQ was suppressed by the expression of the FLAG-DDHD2 WT, but not the S351A mutant or the mutants related to SPG, in WT MEFs (Fig. 5c). These results suggest that DDHD2 is responsible for the prevention of ROS generation.

Next, we examined whether DDHD2 contributes to the removal of formed ROS. *DDHD2* KO MEFs and WT MEFs were incubated with AR for 3 h to allow ROS accumulation, followed by incubation in the absence of AR to monitor ROS consumption. In WT MEFs, the CellROX staining was decreased by 66% at 3 h after AR washout, whereas only 18% reduction was observed for *DDHD2* KO MEFs (Fig. 5d), suggesting that DDHD2 contributes to both the prevention of ROS formation and removal of formed ROS.

### LDs are not accumulated in *DDHD2* KO MEFs

A previous study demonstrated the accumulation of LDs in the brain of DDHD KO mice^22^. We therefore examined whether *DDHD2* KO MEFs also contain increased levels of LDs and TAG. However, LipiDye staining showed that the numbers of LDs were not significantly different between WT and *DDHD2* KO MEFs (Supplementary Figure 5a). Similarly, no significant difference in the number of LDs was observed between WT and *DDHD2* KO MEFs when LD formation was induced by oleic acid (OA). The TAG content was also indistinguishable between WT and *DDHD2* KO MEFs regardless of whether LD formation was induced by OA or not (Supplementary Figure 5b). These results indicate that the formation of ROS in *DDHD2* KO MEFs is not due to the accumulation of neutral lipids.

### Reaction of lipid peroxidation sensors in DDHD2 MEFs

ROS formation causes lipid peroxidation^29^. We monitored possible lipid peroxidation using two fluorescence sensors, MitoPeDPP^30^ and BODIPY 581/591 C11^31^. Both sensors detected lipid peroxidation in *DDHD2* KO MEFs (Supplementary Figures 6a and b). The increased MitoPeDPP staining was suppressed by the expression of DDHD2-WT-mCherry, but not the S351A mutant (Supplementary Figure 6c). To determine whether possibly oxidized lipids can be removed by DDHD2, WT and *DDHD2* KO MEFs were incubated with 1 mM *tert*-butyl hydroperoxide (t-BHP) for 30 min to allow the accumulation of oxidized lipids, followed by incubation in the absence of t-BHP to monitor degradation of the oxidized lipids formed. In WT MEFs, BODIPY 581/591 C11 green fluorescence reflecting possible lipid peroxidation had decreased by 58% at 1 h after t-BHP washout, whereas no substantial decrease in green fluorescence was observed in *DDHD2* KO MEFs, suggesting that DDHD2 can cleave possibly oxidized lipids (Supplementary Figure 6d; Supplementary Figure 7).

When lipid peroxidation occurs, 4-hydroxynonenal (4-HNE) and malondialdehyde (MDA) are formed as degradation products of oxidized lipids^29^. We therefore examined by WB lysates of spinal cords of 6-month-old *DDHD2* KO mice and detected 4-HNE-modified proteins (Supplementary Figure 6e). Similarly, 4-HNE-modified proteins were detected in lysates of *DDHD2* KO MEFs (Supplementary Figure 6f). Moreover, an enhanced level of MDA was observed in brain lysates of 6-month-old *DDHD2* KO mice (Supplementary Figure 6g).

### Decreased cardiolipin (CL) levels in *DDHD2* KO MEFs

**Fig. 6:**
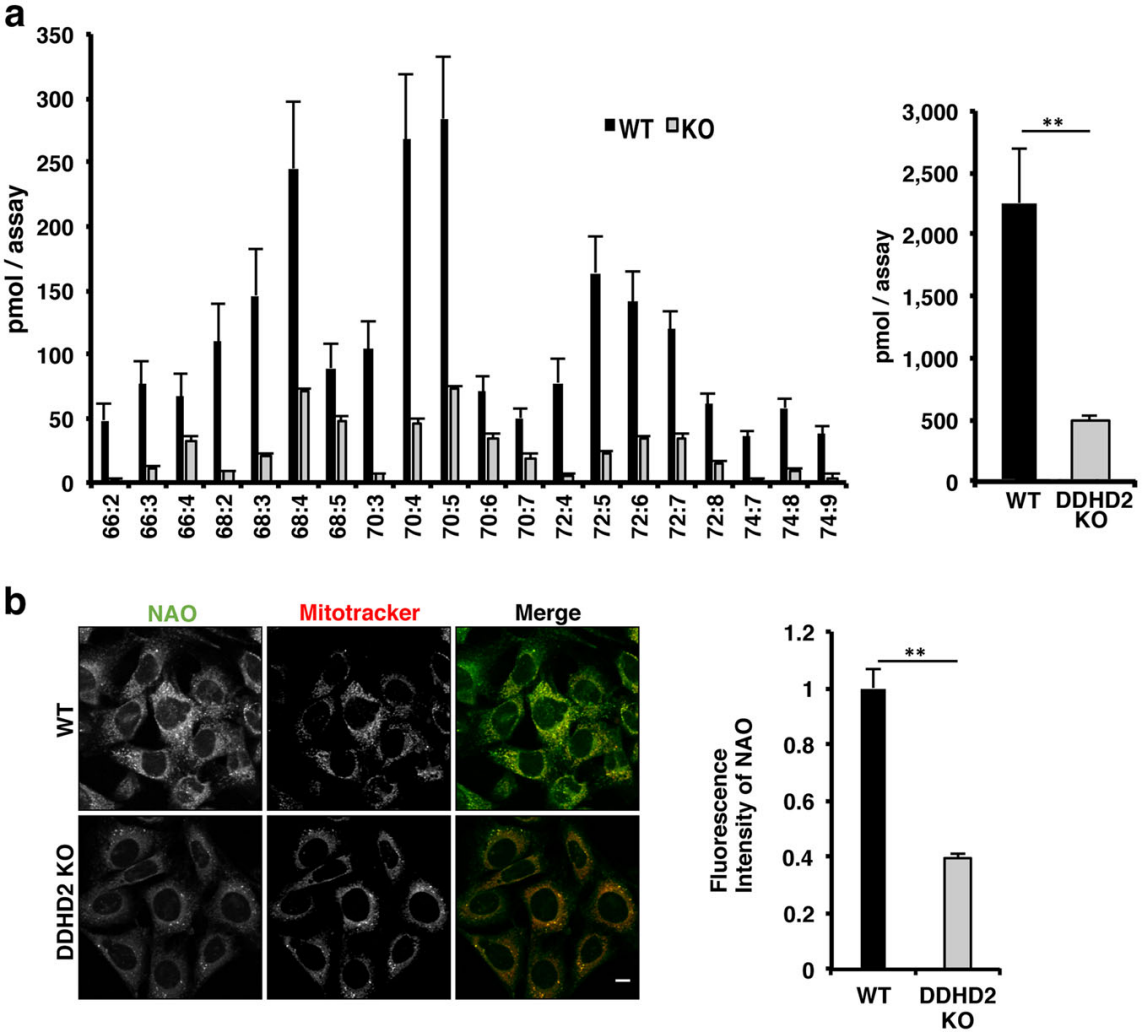
Reduction of CLs in *DDHD2* KO MEFs. **a** The CL species in WT and *DDHD2* KO MEFs were analyzed by mass spectrometry, as described under Materials and methods. The bar graph shows the total amounts of CLs. **b** WT and *DDHD2* KO MEFs were incubated with 1 µM NAO and 500 nM MitoTracker Red FM for 30 min, and then analyzed by IF microscopy. The scale bar is 10 μm. The fluorescence intensity of NAO was quantified and is expressed as the ratio relative to that of WT MEFs. Data in this figure represent means ± SEM (*N* = 3). **P < 0.01, Student’s *t*-test

When MEFs were subjected to lipid analysis by mass spectrometry, unexpectedly we did not detect oxidized phospholipids but found a substantial decrease in CL content (Fig. 6a). Some other phospholipids including phosphatidylcholine appeared to be slightly decreased, but not significant (data not shown). Consistent with the results of mass spectrometric analysis, the staining intensity of *DDHD2* KO MEFs with 10-*N*-nonyl acridine orange (NAO), a CL-specific probe commonly used to monitor CL levels in live cells^32^, was lower than that of WT MEFs (Fig. 6b).

### DDHD2 localizes to ROS-producing mitochondria

**Fig. 7:**
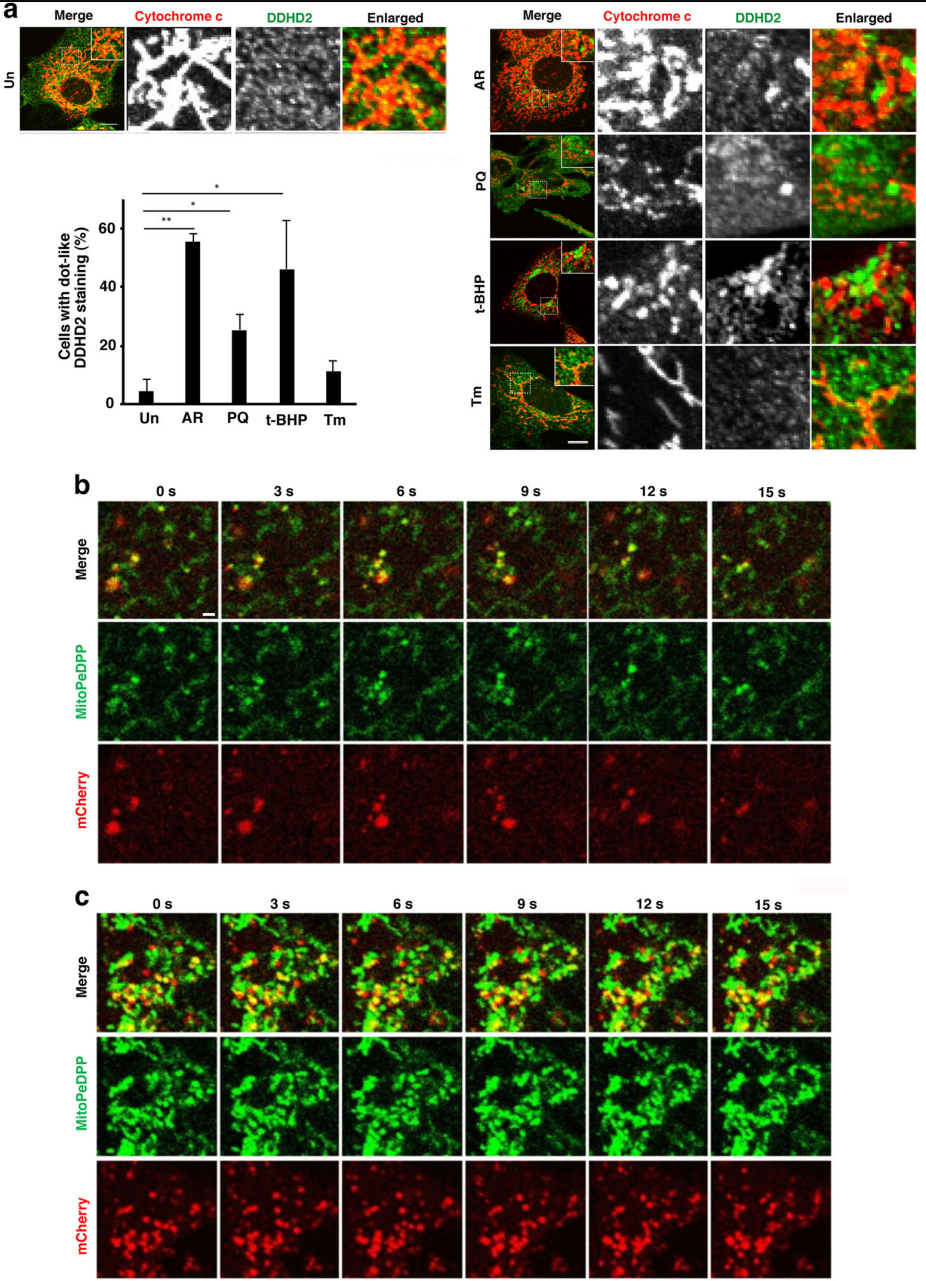
DDHD2 partially localizes to mitochondria and becomes aggregated in response to oxidative stress. **a** WT MEFs were untreated (Un) or treated with 2 µM antimycin A and 1 µM rotenone (AR), 0.1 mM PQ, 0.1 mM t-BHP or 1 µg/ml tunicamycin (Tm) for 2 h, followed by immunostaining with antibodies against DDHD2 and cytochrome c. The number of cells with DDHD2 dots was determined. Data represent means ± SEM (*N* = 3). *P < 0.05, **P < 0.01, Student’s *t*-test. The scale bars are 10 μm. **b**, **c** *DDHD2* KO MEFs with stable expression of DDHD2-WT-mCherry or DDHD2-S351A-mCherry were treated with 0.1 mM t-BHP for 30 min, and then incubated with 0.1 µM MitoPeDPP for 15 min and analyzed by time-lapse imaging. The scale bar is 1 μm. See Supplementary Movies 1 and 2

Our previous study demonstrated that DDHD2 localizes to the ER, Golgi apparatus, and cytosol^7,19,20^. Upon treatment of cells with CI-976, a lysophospholipid acyltransferase antagonist, DDHD2 was found to be redistributed to tubular structures in close vicinity to mitochondria^21^. Given that DDHD2 contributes to the removal of mitochondrial ROS and oxidized lipids, we explored the possibility that DDHD2 interacts with mitochondria. On immunofluorescence (IF) microscopy, DDHD2 in MEFs exhibits a dispersed pattern (Fig. 7a), and this staining was abolished upon *DDHD2* KO (data not shown), suggesting the specificity of the antibody used. When MEFs were incubated with reagents that cause oxidative stress, DDHD2 formed large aggregates (Fig. 7a, right, top, second, and third rows), whereas such aggregation was not obvious upon treatment with tunicamycin, an ER stress inducer (bottom row).

We performed live cell imaging using DDHD2-WT-mCherry and the S351 mutant expressed in *DDHD2* KO MEFs. DDHD2-WT-mCherry moved and transiently stayed together with MitoPeDPP (Fig. 7b; Supplementary Movie 1), whereas the S351 mutant appeared to consistently associate with MitoPeDPP with much less movement (Fig. 7c; Supplementary Movie 2). These results suggest that DDHD2 can interact with mitochondria, and that its inactive mutant stays longer compared with WT DDHD2.

## Discussion

In the present study we demonstrated that not only motor neurons but also MEFs from *DDHD2* KO mice cannot proliferate well in vitro. We found that both *DDHD2* KO MEFs and U2OS cells depleted of DDHD2 by siRNAs are susceptible to apoptotic stimuli. Further analysis revealed that loss of DDHD2 causes ROS production in mitochondria. The notion that ROS production is responsible for the enhanced apoptosis sensitivity was corroborated by the finding that NAC prevented apoptosis. Enhanced generation of ROS upon DDHD2 ablation might produce lipid electrophiles, which interfere with protein function through modification, leading to apoptosis of cultured cells and perhaps motor neurons. Expression of WT DDHD2 in DDHD2-depleted cells not only prevented the formation of ROS, but also facilitated its clearance. It is of note that DDHD2 deficiency could not be compensated for by the expression DDHD2 mutants related to SPG, as well as the active-site mutant. In addition, DDHD2 could not be substituted with DDHD1, another member of the PLA_1_ family implicated in SPG28.

Recent studies revealed that ROS production and LD accumulation are closely correlated^33^. In hepatocytes, ROS levels were found to be proportional to LD accumulation^34^. In *Drosophila*, LDs formed in glial cells during oxidative stress accommodate polyunsaturated fatty acids that have been present in the plasma membrane before oxidative stress, which possibly protects polyunsaturated fatty acids from lipid peroxidation by keeping them away from the ROS-sensitive plasma membrane pool^35^. In the case of photoreceptors (neurons), ROS production stimulates the synthesis of lipids in neurons that are subsequently delivered to neighboring glial cells to induce LD accumulation^36^. A subsequent study by the same group suggested that LD accumulation prevents neurodegeneration through scavenging of peroxidized lipids^37^. In *DDHD2* KO mice, LDs were found to accumulate in the brain (ref. ^22^ and this study). This can be explained by the finding that DDHD2 has TAG lipase activity^22^. However, lack of the TAG lipase activity in the brain may not be a sole reason for LD accumulation. Our present results demonstrated that DDHD2-depleted cells generate ROS, raising the possibility that LD accumulation in the brain of *DDHD2* KO mice is partly a consequence of ROS generation. MEFs may express higher levels of TAG lipases than neuronal cells, thereby preventing LD accumulation, even ROS generation. Taken together, ROS generation, as well as LD accumulation, may contribute to the pathogenesis of SPG.

Mass spectrometric analysis revealed that the CL levels are decreased in *DDHD2* KO MEFs. This is in contrast to the case of yeast homolog Ddl1 (Yor022c); loss of the *Ddl1* gene resulted in an increase (**~**21%) in the CL content^38^. Yadav and colleagues^38^ hypothesized that yeast PLA_1_ Ddl1, in cooperation with phospholipase A_2_ Cld1, participates in CL remodeling, and that dilysocardiolipin formed by a sequential cleave by the two enzymes undergo degradation. The reason for these opposite results between yeast and mammals is not known at present, but the present results raise the possibility that DDHD2 also directly or indirectly participates in CL remodeling. CLs play important roles for mitochondrial functions. They are essential for the architecture and activity of mitochondrial respiratory chain complexes^39^. Therefore, reduction of CLs in *DDHD2* KO MEFs can explain the phenotype of cells: ROS production and reduction in ATP production, mitochondrial membrane potential, and respiratory activity.

Mass spectrometric analysis also revealed no marked accumulation of oxidized lipids in *DDHD2* KO MEFs. This is rather surprising because the lipid peroxidation-degradation products 4-HNE and MDA seem to be significantly increased in DDHD2-depleted cells (Supplementary Figures 6e–g). Moreover, positive signals were seen for two lipid peroxidation sensors, MitoPeDPP and a commonly used BODIPY 581/591 C11 (Supplementary Figures 6a and b). One explanation for these apparent inconsistencies is that ROS generation may be massive enough to oxidize lipid sensors, but not endogenous lipids. It has been reported that BODIPY 581/591 C11 is more sensitive to oxidative stress than authentic phospholipids^40^. Alternatively, but not exclusively, peroxidized lipids may promptly undergo nonenzymatic Hock cleavage, producing 4-HNE and MDA.

In conclusion, the present study revealed that DDHD2 plays a role in preventing ROS production and protecting cells against oxidative stress, although the mechanisms for these functions remain obscure. One possibility is that DDHD2 cleaves oxidized lipids to mitigate oxidative stress. This is consistent with the findings that the expression of DDHD2 can prevent the reaction of lipid peroxidation sensors and that DDHD2 forms aggregates upon oxidative stress and moves together with MitoPeDPP-positive mitochondria. In future studies the possibility that DDHD2 cleaves oxidized lipids should be tested. To develop drugs for SPG, *DDHD2* KO mice may be useful. Obviously, antioxidants that block ROS formation are good candidates that may ameliorate the progression of HSP.

## Materials and methods

### Animals

All animal procedures and experiments were approved by the Animal Care Committee of Tokyo University of Pharmacy and Life Sciences, and conducted according to the guidelines of the committee.

### Plasmid construction

The mammalian expression plasmids for FLAG-tagged human DDHD2 and its S351A mutant were described previously^7^. FLAG-tagged human DDHD2 SPG mutants were generous gifts from Dr. Hiroshi Doi (Yokohama City University).

### Retrovirus expression system

The DNA fragments encoding pENTER-tagged human DDHD1, and DDHD2 and its mutants were inserted into the retrovirus vector pMRX-IRES-puro-DEST-mCherry. For the production of recombinant retroviruses, PLAT-E cells were transfected with retrovirus vectors using a PEI MAX reagent according to the manufacturer’s protocol. To establish stable cell lines, cells were infected with recombinant retroviruses and cultured in medium containing 10 µg/ml puromycin.

### RNA interference

The siRNA targeting sequences used were as follows: Luciferase siRNA, CGTACGCGGAATACTTCGA; DDHD2 siRNA#2, AAGAAAGAAGAUAUUAAACUA; and DDHD2 siRNA#3, AAGGAGAAAGUAGAUAAGGAA. U2OS cells were transfected with 100 nM siRNA using Lipofectamine RNAiMAX (Thermo Fisher Scientific) according to the manufacturer’s protocol. Cells were fixed and processed at 72 h after transfection.

### Cell culture and plasmid transfection

MEFs and U2OS cells were grown in DMEM supplemented with 10% fetal calf serum (FCS). Plasmids were transfected using Lipofectamine 3000 (Thermo Fisher Scientific) or Neon (Thermo Fisher Scientific) according to the manufacturer’s protocol.

### Preparation of MEFs and cell culture

MEFs were isolated from E13.5 embryos and immortalized with the SV40 large T antigen. The immortalized cells were cultured in medium containing 2 µg/ml puromycin.

### Antibodies and probes

A monoclonal antibody against 4-HNE (No. ab48506; 1:100 dilution for WB) and a polyclonal antibody against α-tubulin (No. ab4074; 1:100,000 dilution for WB) were obtained from Abcam. A polyclonal antibody against SOD1 (No. HPA001401, 1:1000 for WB), monoclonal antibodies against GFAP (No. G3893; 1:200 dilution for IF), and FLAG (No. F7425; 1:1000 dilution for IF) were obtained from Sigma-Aldrich. A polyclonal antibody against Tom20 (No. FL-145; dilution for 1:1000), monoclonal antibodies against Bax (No. sc-493; 1:200 dilution for IF), and glyceraldehyde 3-phosphate dehydrogenase (GAPDH) (sc-47724; 1:1000 dilution for WB) were purchased from Santa Cruz Biotechnology. Monoclonal antibodies against SMI32 (No. 801701; 1:1000 dilution for IF and WB) and cytochrome c (No. 556432; 1:1000 dilution for IF) were purchased from BioLegend and BD Pharmingen, respectively. A polyclonal antibody against cleaved caspase3 (No. 9664; 1:1000 dilution for IF) was purchased from Cell Signaling. A polyclonal antibody against catalase (No. 219010, 1:1000) was purchased from Merck Millipore. A polyclonal antibody against mouse DDHD2 was raised against a recombinant GST-tagged DDHD2 fragment (amino acids 368–476) and affinity-purified using antigen-coupled beads. MitoTracker Green and Red CMXRos, MitoSOX Red Mitochondrial Superoxide Indicator, CellROX Green reagent, and BODIPY 581/591 C11 were purchased from Thermo Fisher Scientific. MitoPeDPP was purchased from Dojindo.

### Generation of DDHD2 knock-out mice

A DDHD2 gene fragment was isolated from a mouse genomic bacterial artificial chromosome library derived from the 129sv mouse strain (RP22-60F20) and subcloned into pBluescript II SK. A conditional targeting vector was constructed using a highly efficient recombineering-based method^41^. The DDHD2 targeting vector was digested with NotI, and then electroporated into E14 embryonic stem cells. Cells resistant to G418 were picked up and analyzed. Targeted cell lines were injected into C57BL/6J blastocysts to produce chimeric mice. The male chimeric mice were crossed with female WT C57BL/6J mice, by which DDHD2^+/flox^ offspring were produced. To disrupt the DDHD2 gene, DDHD2^+/flox^ offspring were mated with CAG-Cre transgenic mice (RIKEN BRC)^42^, which express Cre recombinase at early stages of development. The DDHD2^−/−^ mice were backcrossed to C57BL/6J for 10 generations.

### Analysis of lower limb motor function in mice

We evaluated the motor function of lower limbs in mice as described previously^43^. For clasping analysis, mice were suspended by the tail above an open cage for 30 s for 10 trials. More than 10 animals were analyzed for the characterization of each phonotype. For the extension reflex test, mice were suspended by the tail for 30 s, and then analyzed using the three-point score system. Score 0: bilateral hind limbs were completely crossed, score 1: bilateral hind limbs were of <90° at an angle as to the vertical plane, score 2: the unilateral hind limbs were of <90° at an angle as to the vertical plane, and score 3: bilateral hind limbs were extended about 120°.

### Immunostaining of frozen sections

Mice were subjected to perfusion fixation with 4% paraformaldehyde in phosphate-buffered saline (PBS). After the perfusion fixation, spinal cords were isolated from the mice, and then sequentially immersed in 10% sucrose, 20% sucrose, and 30% sucrose solutions. The spinal cords were frozen in O.C.T. compound, and then cut with a microtome into 10-µm thick sections. The frozen sections were incubated with 0.2% TritonX-100 in PBS for membrane permeabilization, blocked with 2% bovine serum albumin (BSA) in PBS for 1 h at room temperature, and then incubated with a primary antibody in 2% BSA in PBS overnight at 4 °C. The next day, the frozen sections were incubated with a secondary antibody in 2% BSA in PBS for 45 min at room temperature, mounted on a glass slide and analyzed.

### Hematoxylin and eosin staining of spinal cord sections

Hematoxylin and eosin staining were performed according to the protocols of Abnova. The number of motor neurons in an arbitrarily chosen microscopic field with a size of 124 × 124 μm^2^ was determined. In each mouse, six fields were analyzed, and three mice were used for each experiment.

### Culture of primary motor neurons from spinal cords and brains of embryonic mice

Mice embryos were isolated from pregnant mice at E14.5 or P1. Spinal cords and brains were dissected out and transferred to PBS. The spinal cords and neurons were incubated with a papain/EDTA solution for 10 min at 37 °C and transferred through a 40-µm cell strainer to 50-ml tubes including primary neuron medium (D-MEM/F12 containing 20% FCS, 50 IU/ml penicillin, 50 µg/ml streptomycin, and N2 and B27 supplements). After centrifugation, the precipitated cells were suspended in the medium and plated on six-well plates with glass coverslips precoated with poly-l-lysine (2 × 10^5^ cells/well). After one day, the culture medium was replaced with medium containing 2% FCS. After culture for 2 or 7 days, the cells were fixed and immunostained with an antibody against SMI32 or MAP2.

### Assessment of ROS production

To detect ROS production, cells were incubated with 2.5 µM CellROX Green for 30 min at 37 °C. To detect mitochondrial ROS production, cells were incubated with 5 µM MitoSOX for 10 min.

### Detection of lipid peroxidation

Cells were incubated with 2 µM BODIPY 581/591 C11 for 30 min at 37 °C. The cells were washed with medium prior to live-cell imaging. Fluorescence of peroxidized species (excitation at 473 nm) and non-oxidized species (excitation at 559 nm) was recorded. Alternatively, cells were incubated with 0.1 µM MitoPeDPP for 15 min at 37 °C, and MitoPeDPP fluorescence was detected.

To measure the MDA content, brains from 6-month-old mice were homogenized in HB buffer (10 mM Hepes, 220 mM mannitol, and 0.07 M sucrose, pH 7.4). The homogenate was centrifuged at 800×*g* at 4 °C for 10 min, and then the supernatant was centrifuged at 12,000×*g* at 4 °C for 10 min to pellet mitochondria. The mitochondrial MDA amount was measured using a TBARS assay kit (Cayman: No.10009055) according to the manufacturer’s protocol.

### LD detection in MEFs

Cells were incubated with 1 µM LipiDye (Funakoshi) for 30 min, and then fixed. LipiDye fluorescence was detected.

### TAG measurement

TAG was measured using a triglyceride quantification colorimetric kit (Bio Vision: No. K662) according to the manufacturer’s protocol.

### Measurement of mitochondrial CLs by NAO

Cells were incubated with 1 µM NAO (Thermo Fisher Scientific) for 30 min, and then fixed.

### CL analysis using LC–MS/MS

Lipids were extracted from cultured cells with the Bligh–Dyer method^44^. An aliquot of the lower (organic) phase was evaporated to dryness under N_2_, and the residue was dissolved in methanol for LC/MS/MS measurement. LC–electrospray ionization–MS/MS analysis was performed with a Q-Exactive plus mass spectrometry equipped with an UltiMate 3000 LC system (Thermo Fisher Scientific). Lipid samples (10 μl) were separated on a Waters CORTECS UPLC C18 column (1.6 μm, 2.1 mm × 150 mm i.d.) at 40 °C (column oven) using solvent A (acetonitrile/methanol/water (1/1/2, v/v/v) supplemented with 5 mM ammonium formate and 0.05% ammonium hydroxide) and solvent B (isopropanol/acetonitrile (4/1, v/v) supplemented with 5 mM ammonium formate and 0.05% ammonium hydroxide). The following gradient was used: 0% B (0–2 min), a linear gradient from 0% B to 100% B (2–37 min), 100% B (37–49 min), a linear gradient 100% B to 0% B (49–49.5 min), and 0% B (49.5–60 min). Flow rate was 220 μl/min. CL species were measured by a full scan within *m*/*z* 300–1800 in the negative ion mode, using an Orbitrap Fourier transform mass spectrometry (FTMS) analyzer with a resolution of 70,000. CL (14:0/14:0/14:0/14:0), which is assumed not to occur naturally, was added to samples as an external standard for correction and quantification of CL amount.The mass accuracy was always within 5 ppm. The ion spray voltage was set to −3.2 kV. The heated capillary temperature was set at 300 °C. The other parameters were set according to the manufacturer’s recommendations. The MS system was controlled with the Xcalibur software.

### Measurement of the mitochondrial membrane potential

Cells were incubated with 2 µM JC-1 (5,5′,6,6′-tetrachloro-1,1′,3,3′-tetraethylbenzimidazolylcarbocyanine iodide: Thermo Fisher Scientific) for 30 min at 37 °C, and then washed with medium. Green/Red fluorescence of JC-1 was recorded.

### Measurement of ATP

ATP was measured using a Celltiter-Glo luminescent cell viability assay kit (Promega) according to the manufacturer’s protocol. The ATP amount was normalized as to protein concentration.

### Measurement of OCR of MEFs

Mitochondrial respiratory capacity was measured using an Oxygen Meter Model 781 equipped with a Mitocell MT200 (a closed respiratory chamber) (Strathkelvin Instruments). WT and *DDHD2* KO MEFs were cultured in DMEM supplemented with 10% FCS. The cells were then trypsinized and resuspended at a density of 4.0–8.0 × 10^6^ cells/ml. Basal respiration was measured in DMEM medium in the presence of 1 mM pyruvate and 24 mM glucose at 37 °C without CO_2_ supply. MEFs were subsequently incubated with 1 µg/µL oligomycin and 4 µM carbonyl cyanide 4-(trifluoromethoxy)phenylhydrazone (FCCP) to measure OCR under conditions blocking ATP synthesis and maximal respiration, respectively.

### IF microscopy

For IF microscopy, cells were fixed with 4% paraformaldehyde in PBS for 20 min at room temperature, and then permeabilized with 0.2% Triton X-100 in PBS and blocked with 2% BSA in PBS. After blocking, the cells were incubated with primary, and then secondary antibodies in 2% BSA in PBS.

### Time-lapse imaging

Live-cell imaging was performed using an Olympus FluoView 1000 laser scanning microscope. *DDHD2* KO MEFs stably expressing DDHD2-WT-mCherry or DDHD2-S351A-mCherry were plated on a glass-bottom dish. The next day, the cells were treated with 0.1 mM t-BHP for 30 min, and then incubated with 0.1 µM MitoPeDPP for 15 min. The dish was mounted on the stage of the microscope with a stage-top incubator (37 **°**C, 5% CO_2_), and live-cell imaging was performed. Time-lapse images were captured at 1 s intervals for 10 min.

### Statistical analysis

All experiments were performed at least three times. For quantification of fluorescence intensity, at least 30 cells were examined. ImageJ was used for quantification. All results are expressed as means ± SEM. Statically significant differences were determined using Student’s *t*-test. Differences were considered to be significant if the *P*-value was <0.05.

## Electronic supplementary material

Movie S1

Movie S2

Supplemental material
