# Supplementary material for: Loss of DDHD2, whose mutation causes spastic paraplegia, promotes reactive oxygen species generation and apoptosis
Source: Cell Death Dis. 2018 Jul 23;9(8):797. doi: 10.1038/s41419-018-0815-3 (PMC6056544; doi:10.1038/s41419-018-0815-3)

Supplementary Figure Legends

Supplementary Figure 1 *DDHD2* KO mice exhibit a phenotype similar to HSP. (**a**) Schematic representation of the Cre-mediated DDHD2 deletion strategy. The lipase domain is encoded in exons 8 and 9. Cre-mediated deletion of exons 8 and 9 is supposed to cause a frame shift. Neo^r: neomycin resistance gene, FRT: Flp recombination target. (**b**) Southern blot analysis of BglI-digested genomic DNA derived from WT (+), hetero (+/-), and knockout (-/-) alleles using the 3' probe depicted in (**a**). WB analysis of testis lysates with antibodies against DDHD2 and α-tubulin. (**c**) Hind limb clasping of WT and *DDHD2* KO mice. Determination of the number of clasping responses during 10 tail suspensions in WT and *DDHD2* KO mice at 1-12 months of age ($N \geq 10$). (**d**) Quantification of the hind limb extension reflex in WT and *DDHD2* KO mice at 1-12 months of age ($N \geq 10$). (**e**) Determination of the stride length of the hind limb in WT and *DDHD2* KO mice at 6 months of age (N=3). (**f**) Primary motor neurons from the motor cortex were cultured for 7 days, and then immunostained with an anti-MAP2 antibody. Scale bar, 10 μm. The number of surviving MAP2-positive motor neurons in a microscopic field (212 X 212 μm^2) was determined. The bar graph shows the number of MAP2-positive neurons (N=3). No significant difference in viability was observed between WT and *DDHD2* KO neurons. Data in this figure represent means ± S.E.M. *P < 0.05, **P < 0.01, Student's t-test.

Supplementary Figure 2 Knockdown of DDHD2 in U2OS cells increases apoptosis sensitivity. U2OS cells were transfected with luciferase (control) siRNA, DDHD2 siRNA#2, or DDHD2 siRNA#3. At 72 h after transfection, the cells were treated with 1 μM STS for 24 h (**a**) or 60 μM H_2O_2 for 24 h (**b**), and then subjected to TUNEL staining or immunostaining with an anti-cleaved caspase3 antibody, respectively. The number of

TUNEL-positive cells (**a**) or cleaved caspase3-positive cells (**b**) was determined. The scale bars for whole cell panels are 10 μm. Data represent means ± S.E.M. (*N*=3). *P < 0.05, **P < 0.01, Student's t-test.

Supplementary Figure 3 Knockdown of DDHD2 in U2OS cells induces ROS formation and mitochondrial dysfunction. (**a**) U2OS cells transfected with luciferase (control) siRNA or DDHD2 siRNA#2 were incubated with 2.5 μM CellROX Green for 30 min, and then analyzed by IF microscopy. The fluorescence intensity of CellROX Green was quantified and is expressed as the ratio relative to that in control cells. (**b**) U2OS cells transfected with luciferase (control) siRNA, DDHD2 siRNA#2, or DDHD2 siRNA#3 were incubated with 2.5 μM MitoSOX and 500 nM MitoTracker Green FM for 10 min, and then analyzed without fixation. The fluorescence intensity of MitoSOX was quantified and is expressed as the ratio relative to that in control cells. (**c**) U2OS cells transfected with luciferase (control) siRNA or DDHD2 siRNA#2 were treated or untreated with 200 μM NAC for 24 h, and then immunostained with an anti-cleaved caspase3 antibody. Of note is that cleaved caspase3 was detected in the nucleus in some types of cells[45]. The fluorescence intensity of cleaved caspase3 staining was quantified and is expressed as the ratio relative to that in NAC-untreated control cells. The scale bars for whole cell panels are 10 μm. Data in this figure represent means ± S.E.M. (*N*=3). *P < 0.05, **P < 0.01, Student's t-test.

Supplementary Figure 4 DDHD1 can not compensate for DDHD2 ablation in terms of ROS removal. *DDHD2* KO MEFs with stable expression of mCherry (control), DDHD1-mCherry, or DDHD1-S537A-mCherry were incubated with 2.5 μM CellROX Green for 30 min, and then analyzed by IF microscopy. The scale bar is 10 μm. The

fluorescence intensity of CellROX Green was quantified and is expressed as the ratio relative to that in mCherry-expressing cells. No significant difference was observed between WT and *DDHD2* KO MEFs. Data represent means ± S.E.M. (*N*=3).

Supplementary Figure 5 *DDHD2* ablation does not affect LD formation. (**a**) WT and *DDHD2* KO MEFs were untreated or treated with 150 μM OA. After 16 h, the cells were fixed and stained with LipiDye. The number of LDs per cells was determined. No significant difference was observed between WT and *DDHD2* KO MEFs. The scale bar is 10 μm. (**b**) The amount of TAG was determined and expressed as the ratio relative to that in OA-untreated WT MEFs. No significant difference was observed between WT and *DDHD2* KO MEFs. Data in this figure represent means ± S.E.M. (*N*=3).

Supplementary Figure 6 Loss of DDHD2 causes the oxidization of lipid peroxidation sensors in mitochondria. (**a**) To monitor possibly oxidized lipids, WT and *DDHD2* KO MEFs were incubated with 0.1 μM MitoPeDPP for 15 min, and then analyzed by IF microscopy without fixation. The fluorescence intensity of MitoPeDPP was quantified and is expressed as the ratio relative to that in WT cells. (**b**) WT and *DDHD2* KO MEFs were incubated with 2 μM BODIPY 581/591 C11 for 30 min, and then analyzed by IF microscopy without fixation. The graph shows the normalized ratio of the BODIPY 581/591 C11 Green/Red intensity in *DDHD2* KO cells to that in WT cells. (**c**) *DDHD2* KO MEFs and ones with stable expression of DDHD2-WT-mCherry or DDHD2-S351A-mCherry were incubated with 0.1 μM MitoPeDPP for 15 min, and then analyzed. The fluorescence intensity of MitoPeDPP was quantified and is expressed as the ratio relative to that in *DDHD2* KO cells. (**d**) WT and *DDHD2* KO MEFs were untreated or incubated with 1 mM t-BHP for 30 min, or incubated with 1

mM t-BHP for 30 min, and then 60 min without t-BHP. Cells were incubated with 2 μM BODIPY 581/591 C11 for 30 min, and then analyzed. The graph shows the normalized ratio of the BODIPY 581/591 C11 Green/Red intensity of *DDHD2* KO cells to that in WT cells without t-BHP treatment. (**e**) Lysates (20 μg) of spinal cords of WT and *DDHD2* KO mice at 6 months of age were analyzed by WB with antibodies against DDHD2, 4-HNE and α-tubulin. (**f**) Lysates (30 μg) of WT MEFs, *DDHD2* KO MEFs and WT MEFs treated with 1 mM t-BHP for 1 h were analyzed by WB. (**g**) Accumulation of MDA, a degradation product of oxidized lipids, was determined by TBARS assay. Data in this figure represent means ± S.E.M. (N=3). *P < 0.05, **P < 0.01, Student's t-test. The scale bars for whole cell panels are 10 μm.

Supplementary Figure 7 Peroxidation of lipid sensors does not decrease in *DDHD2* KO MEFs. BODIPY 581/591 staining images related to Supplementary Figure 6d. For details, see the legend to Supplementary Figure 6d. Scale bars, 10 μm.

Supplementary Movie Captions

Supplementary Movie 1 Movement of DDHD2-WT-mCherry relative to oxidized lipids. For details, see the legend to Figure 7b. Images were captured every 3 s.

Supplementary Movie 2 Movement of DDHD2-S351A-mCherry relative to oxidized lipids. For details, see the legend to Figure 7c. Images were captured every 1 s.

Supplementary Figure 1

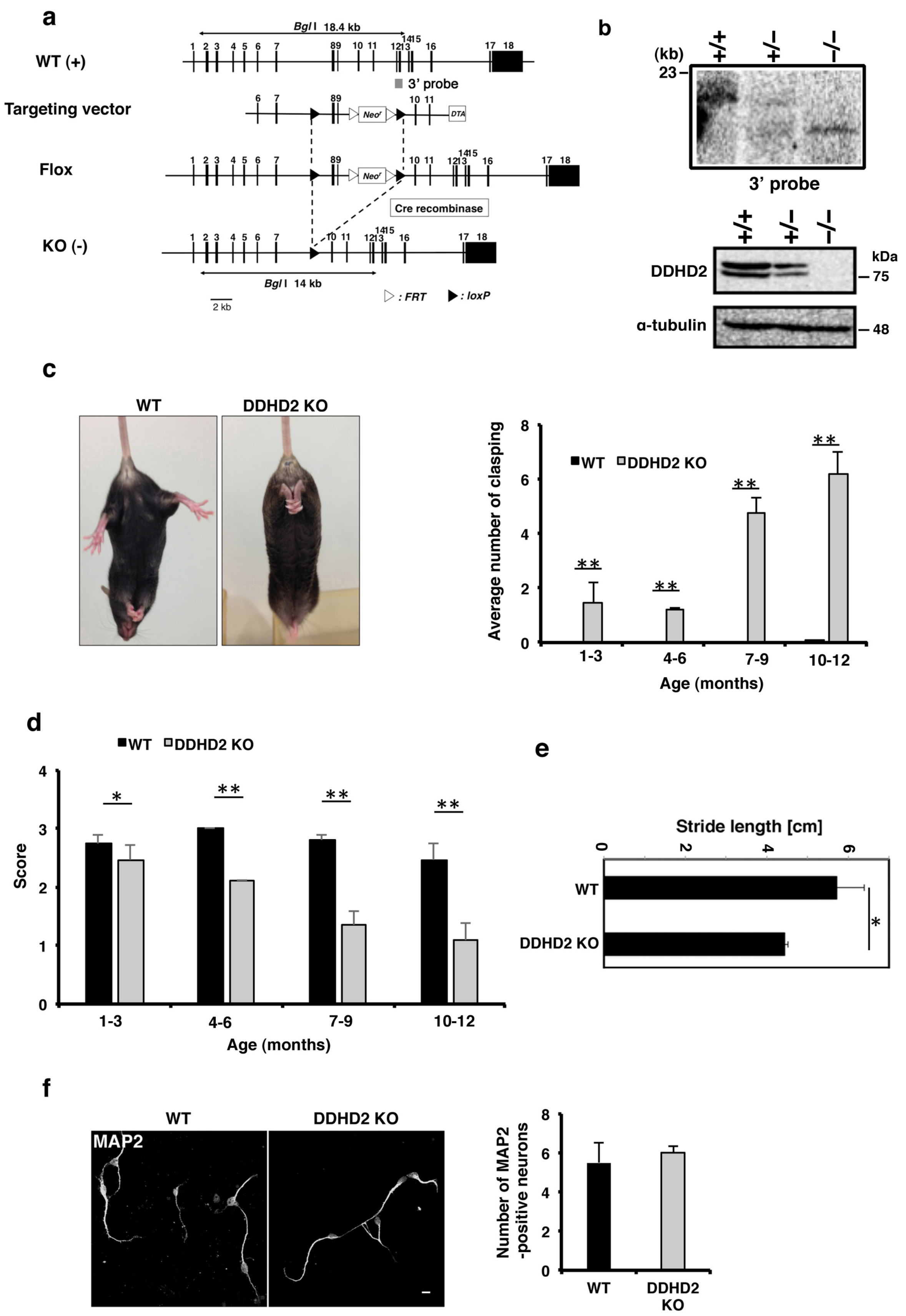

Supplementary Figure 2

a

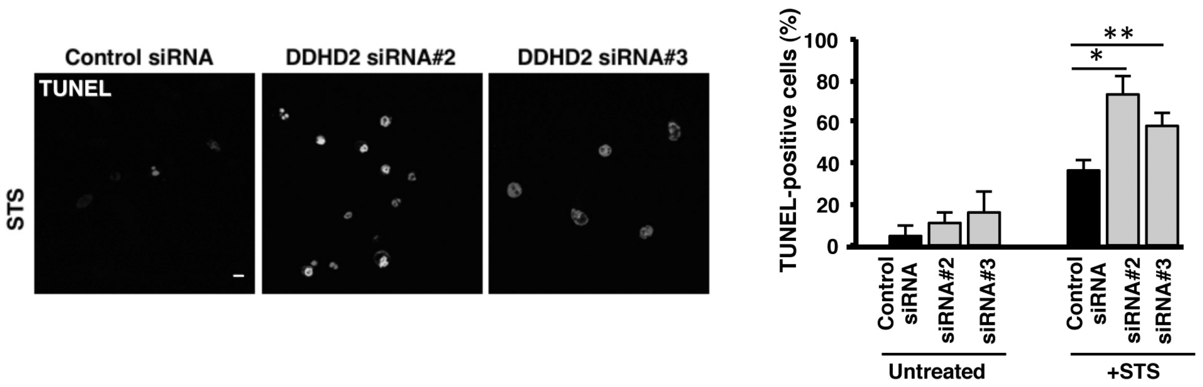

b

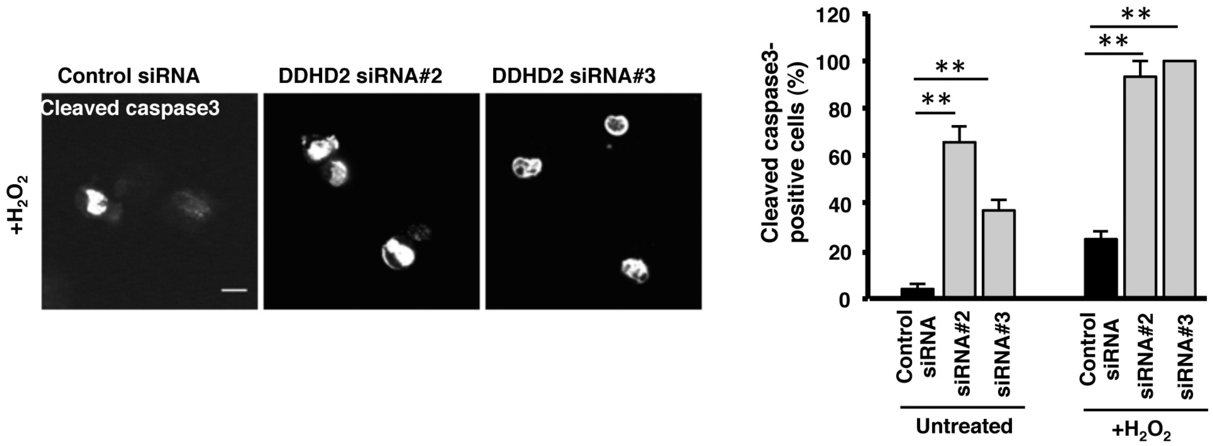

Supplementary Figure 3

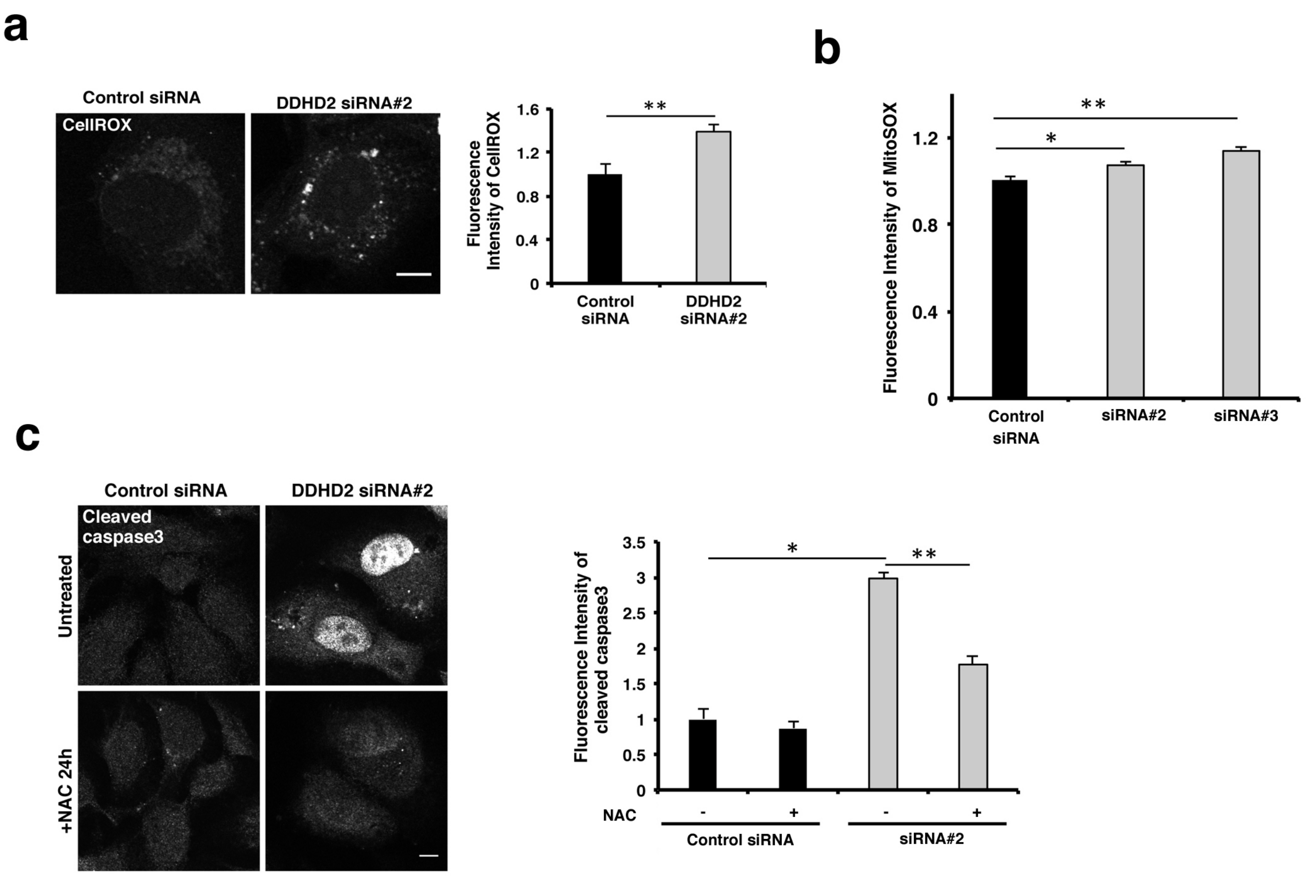

Supplementary Figure 4

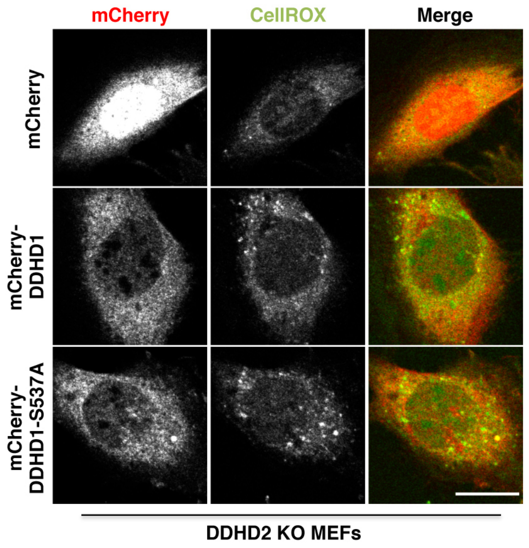

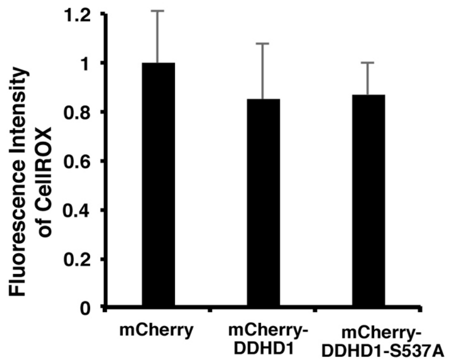

Supplementary Figure 5

a

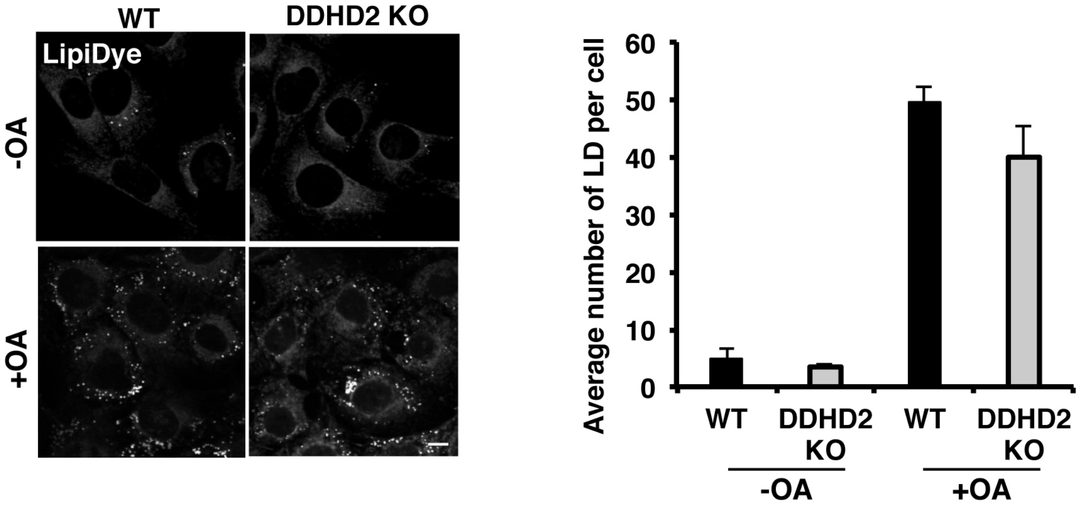

b

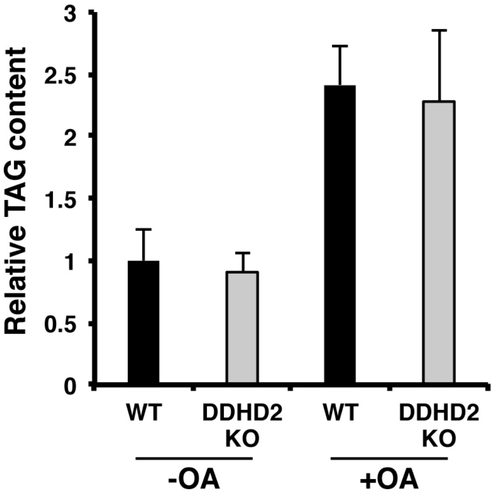

Supplementary Figure 6

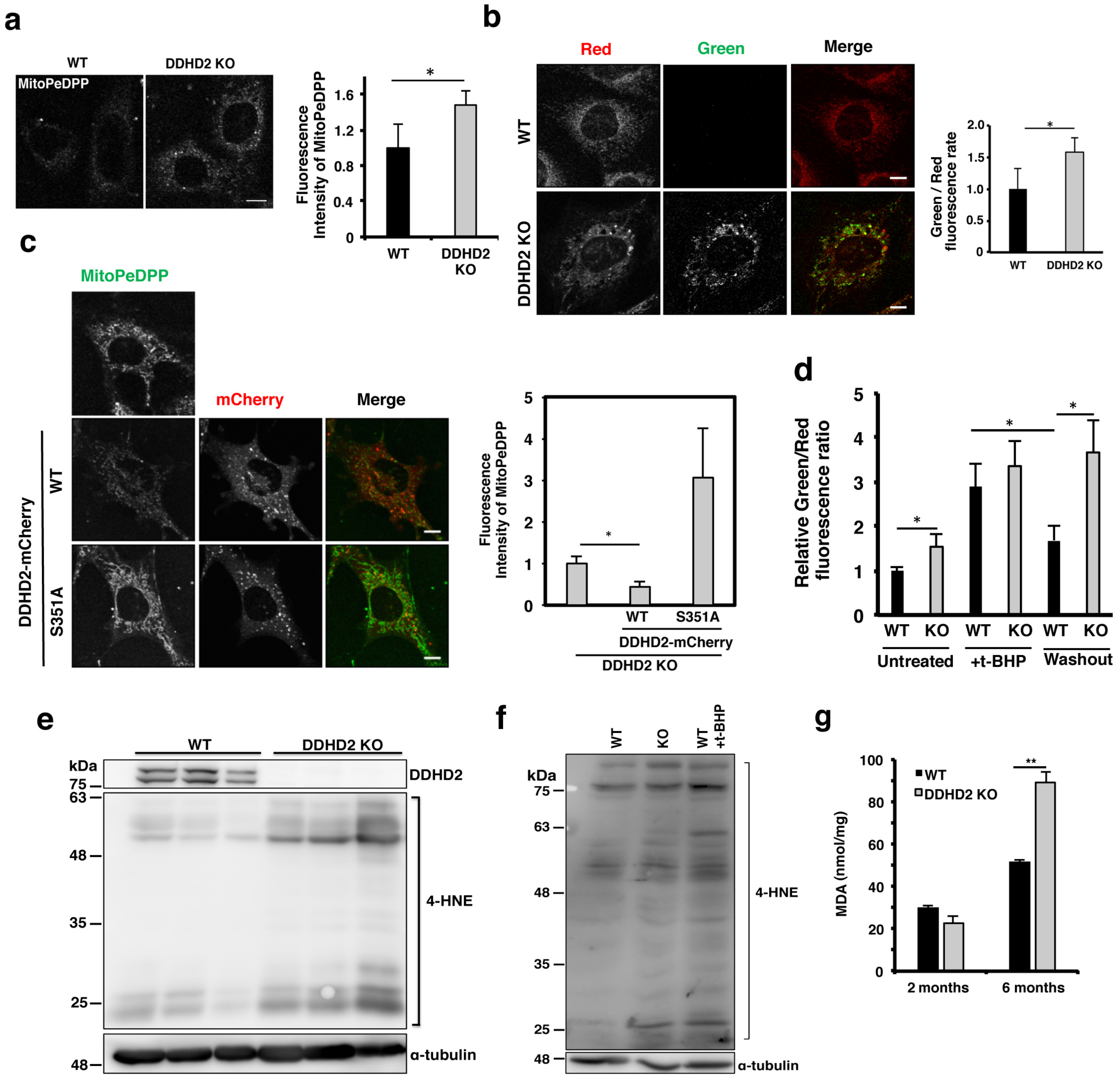

Supplementary Figure 7

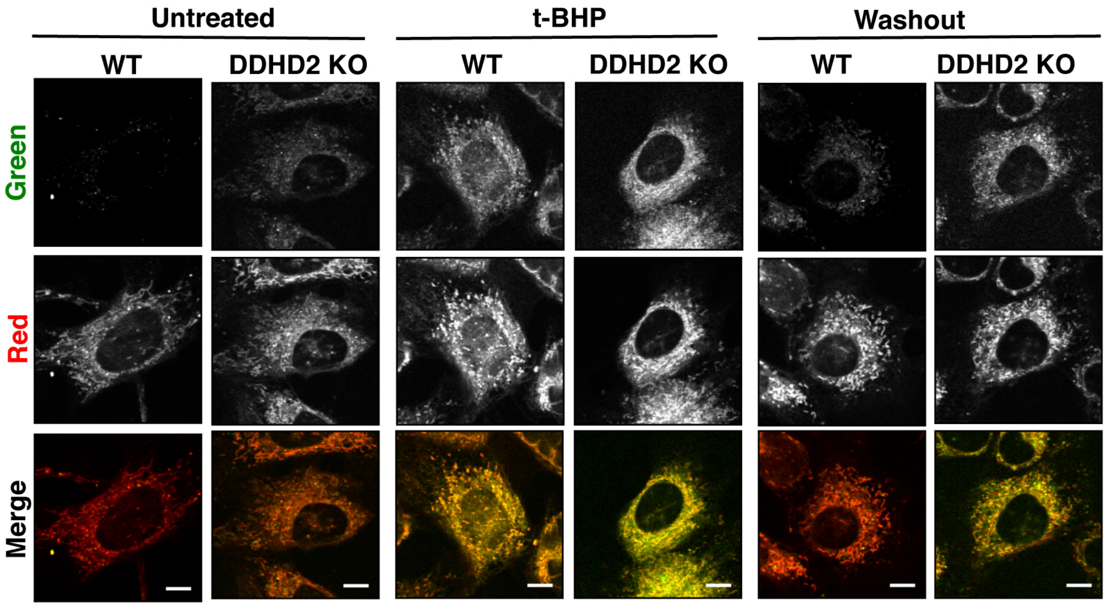

Supplement: Supplementary file 3 — Supplemental material [file 41419_2018_815_MOESM3_ESM.pdf]
